# (5*S*)-3-Chloro-4-(2,5-dihydro-1*H*-pyrrol-1-yl)-5-[(1*R*,2*S*,5*R*)-2-isopropyl-5-methyl­cyclo­hex­yloxy]furan-2(5*H*)-one

**DOI:** 10.1107/S1600536810051226

**Published:** 2010-12-11

**Authors:** Jian-Hua Fu, Zhao-Yang Wang, Fu-Ling Xue, Jing-Pei Huo

**Affiliations:** a School of Chemistry and Environment, South China Normal University, Guangzhou 510006, People’s Republic of China

## Abstract

The title compound, C_18_H_26_ClNO_3_, was obtained *via* a tandem asymmetric Michael addition–elimination reaction of 3,4-dichloro-5-(*S*)-(l-menth­yloxy)furan-2(5*H*)-one and 2,5-di­hydro-1*H*-pyrrole in the presence of potassium fluoride. In the mol­ecule, the nearly planar dihydro­pyrrole ring [maximum atomic deviation = 0.019 (3) Å] is oriented at a dihedral angle of 10.73 (8)° to the the nearly planar furan­one ring [maximum atomic deviation = 0.011 (2) Å]; the cyclo­hexane ring adopts a chair conformation. In the crystal, mol­ecules are linked *via* weak inter­molecular C—H⋯O hydrogen bonds, forming supra­molecular chains running along the *b* axis.

## Related literature

The title compound is a derivative of 4-amino-2(5*H*)-furan­one. For the biological activity of 4-amino-2(5*H*)-furan­ones, see: Lattmann *et al.* (1999 ▶, 2005 ▶, 2006 ▶); Rowland *et al.* (2007 ▶); Kim *et al.* (2002 ▶). For asymmetric Michael addition reactions of 2(5*H*)-furan­one, see: He *et al.* (2006 ▶). For the synthesis of the title compound, see: Song *et al.* (2009 ▶).
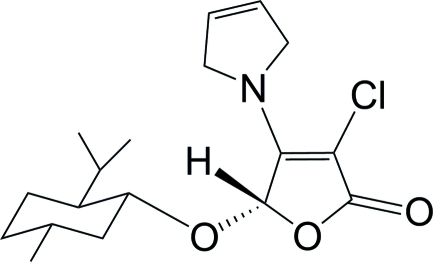


## Experimental

### 

#### Crystal data

C_18_H_26_ClNO_3_
*M* *_r_* = 339.85
Orthorhombic, 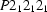

*a* = 7.192 (2) Å
*b* = 9.622 (3) Å
*c* = 27.534 (9) Å
*V* = 1905.4 (10) Å^3^
*Z* = 4
Mo *K*α radiation
μ = 0.21 mm^−1^
*T* = 298 K
0.23 × 0.20 × 0.16 mm

#### Data collection

Bruker APEXII area-detector diffractometer
Absorption correction: multi-scan (*SADABS*; Sheldrick, 1996 ▶) *T*_min_ = 0.952, *T*_max_ = 0.966
9627 measured reflections
3363 independent reflections
1708 reflections with *I* > 2σ(*I*)
*R*_int_ = 0.053

#### Refinement

*R*[*F* ^2^ > 2σ(*F* ^2^)] = 0.052
*wR*(*F* ^2^) = 0.129
*S* = 1.02
3363 reflections
211 parameters
H-atom parameters constrained
Δρ_max_ = 0.11 e Å^−3^
Δρ_min_ = −0.14 e Å^−3^
Absolute structure: Flack (1983 ▶), 1398 Friedel pairs
Flack parameter: 0.14 (12)

### 

Data collection: *APEX2* (Bruker, 2008 ▶); cell refinement: *SAINT* (Bruker, 2008 ▶); data reduction: *SAINT*; program(s) used to solve structure: *SHELXS97* (Sheldrick, 2008 ▶); program(s) used to refine structure: *SHELXL97* (Sheldrick, 2008 ▶); molecular graphics: *ORTEP-3 for Windows* (Farrugia, 1997 ▶); software used to prepare material for publication: *SHELXL97*.

## Supplementary Material

Crystal structure: contains datablocks global, I. DOI: 10.1107/S1600536810051226/xu5106sup1.cif

Structure factors: contains datablocks I. DOI: 10.1107/S1600536810051226/xu5106Isup2.hkl

Additional supplementary materials:  crystallographic information; 3D view; checkCIF report

## Figures and Tables

**Table 1:** Hydrogen-bond geometry (Å, °)

*D*—H⋯*A*	*D*—H	H⋯*A*	*D*⋯*A*	*D*—H⋯*A*
C16—H16⋯O2^i^	0.93	2.42	3.320 (6)	164

Symmetry code: (i) 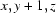.

**Fig. 1.**
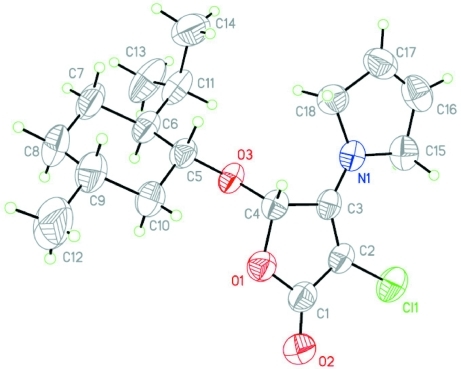
The molecular structure of the title compound showing the atom-labelling scheme. Ellipsoids are drawn at the 50% probability level.

**Fig. 2.**
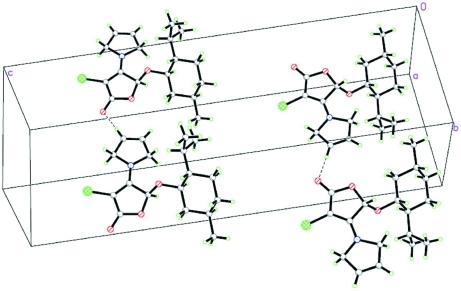
Perspective view of the crystal packing.

## supplementary crystallographic information

### Comment

Molecules possessing 2(5H)-furanone moiety, are extremely useful heterocyclic compounds, due to their significant biological activities, such as antibacterial, anti-inflammatory and antitumor. (Lattmann et al., 2005; Rowland et al., 2007; Kim et al., 2002). 5-Alkoxy-3,4-dihalo-2(5H)-furanones, being a kind of synthons, are widely used in asymmetric and pharmaceutical synthesis reactions. (Lattmann et al., 2006). At the same time, 4-amino-2(5H)-furanones are showing an antibiotic activity against MRSA (Lattmann et al., 1999; Lattmann et al., 2006). Therefore, we are interested in the tandem Michael addition-elimination reaction of the chiral synthon 3,4-dichloro-5-(S)-(l-menthyloxy)-2(5H)-furanone and 2,5-dihydro-1H-pyrrole in the present of potassium fluoride.

The structure of the title compound (I) is illustrated in Fig. 1. The crystal structure of the title compound, which has four chiral centers (C4(S), C5(R), C6(S), C9(R)), contains a five-membered furanone ring and a six-membered ring connected each other via C4—O3—C5 ether bond. The furanone ring of C4—O1—C1—C2—C3 is approximately planar, whereas the six-membered ring displays a chair conformation.

### Experimental

The precursor 3,4-dichloro-5-(S)-(l-menthyloxy)-2(5H)-furanone was prepared according to the literature procedure (Song et al., 2009). After the mixture of 3,4-dichloro-5-(S)-(l-menthyloxy)-2(5H)-furanone (2.0 mmol) and potassium fluoride (6.0 mmol) was dissolved in absolute tetrahydrofuran (2.0 mL) under nitrogen atmosphere, tetrahydrofuran solution of 2,5-dihydro-1H-pyrrole (2.0 mmol) was added. The reaction was carried out under the stirring at room temperature for 6 h. Once the reaction was complete, the solvents were removed under reduced pressure. The residual solid was dissolved in dichloromethane. Then the combined organic layers from extraction were concentrated under reduced pressure, and the crude product was purified by silica gel column chromatography with the gradient mixture of petroleum ether and ethyl acetate to give the product yielding (I) 0.561 g (82.4%).

### Refinement

H atoms were positioned in calculated positions with C—H = 0.93-0.98 Å and were refined using a riding model, with U_iso_(H) = 1.5U_eq_(C) for methyl and 1.2U_eq_(C) for the others.

### Crystal data

**Table:**

C_18_H_26_ClNO_3_	*F*(000) = 728
*M**_r_* = 339.85	*D*_x_ = 1.185 Mg m^−^^3^
Orthorhombic, *P*2_1_2_1_2_1_	Mo *K*α radiation, λ = 0.71073 Å
Hall symbol: P 2ac 2ab	Cell parameters from 1222 reflections
*a* = 7.192 (2) Å	θ = 2.2–17.5°
*b* = 9.622 (3) Å	µ = 0.21 mm^−^^1^
*c* = 27.534 (9) Å	*T* = 298 K
*V* = 1905.4 (10) Å^3^	Block, colourless
*Z* = 4	0.23 × 0.20 × 0.16 mm

### Data collection

**Table:**

Bruker APEXII area-detector diffractometer	3363 independent reflections
Radiation source: fine-focus sealed tube	1708 reflections with *I* > 2σ(*I*)
graphite	*R*_int_ = 0.053
φ and ω scan	θ_max_ = 25.0°, θ_min_ = 2.2°
Absorption correction: multi-scan (*SADABS*; Sheldrick, 1996)	*h* = −6→8
*T*_min_ = 0.952, *T*_max_ = 0.966	*k* = −10→11
9627 measured reflections	*l* = −32→28

### Refinement

**Table:**

Refinement on *F*^2^	Secondary atom site location: difference Fourier map
Least-squares matrix: full	Hydrogen site location: inferred from neighbouring sites
*R*[*F*^2^ > 2σ(*F*^2^)] = 0.052	H-atom parameters constrained
*wR*(*F*^2^) = 0.129	*w* = 1/[σ^2^(*F*_o_^2^) + (0.0439*P*)^2^] where *P* = (*F*_o_^2^ + 2*F*_c_^2^)/3
*S* = 1.02	(Δ/σ)_max_ < 0.001
3363 reflections	Δρ_max_ = 0.11 e Å^−^^3^
211 parameters	Δρ_min_ = −0.14 e Å^−^^3^
0 restraints	Absolute structure: Flack (1983), 1398 Friedel pairs
Primary atom site location: structure-invariant direct methods	Flack parameter: 0.14 (12)

### Special details

**Table:**

Experimental. Data for (I): [α]^20°^_D_ = -45.0° (c 0.557, CH_3_CH_2_OH); ^1^H NMR (400 MHz, CDCl_3_, TMS): 0.791 (3H, *d*, *J* = 6.8 Hz, CH_3_), 0.823-0.937 (7H, *m*, CH, 2CH_3_), 0.945-1.181 (2H, *m*, CH_2_), 1.298-1.457 (2H, *m*, 2CH), 1.623-1.704 (2H, *m*, CH_2_), 2.137-2.210 (2H, *m*, CH_2_), 3.576-3.641 (1H, *ddd*, *J* = 4.4 Hz, *J* = 4.4 Hz,*J* = 4.4 Hz,CH), 3.997-4.970 (4H, *m*, 2CH_2_), 5.731-5.884 (3H, *m*, 3CH), ESI-MS, *m*/*z* (%): Calcd for C_18_H_27_ClNO_3_^+^([M+H]^+^): 340.16(100.0), 342.16(32.7), Found: 340.24 (100.0), 342.32(38.7).
Geometry. All esds (except the esd in the dihedral angle between two l.s. planes) are estimated using the full covariance matrix. The cell esds are taken into account individually in the estimation of esds in distances, angles and torsion angles; correlations between esds in cell parameters are only used when they are defined by crystal symmetry. An approximate (isotropic) treatment of cell esds is used for estimating esds involving l.s. planes.
Refinement. Refinement of F^2^ against ALL reflections. The weighted R-factor wR and goodness of fit S are based on F^2^, conventional R-factors R are based on F, with F set to zero for negative F^2^. The threshold expression of F^2^ > 2sigma(F^2^) is used only for calculating R-factors(gt) etc. and is not relevant to the choice of reflections for refinement. R-factors based on F^2^ are statistically about twice as large as those based on F, and R- factors based on ALL data will be even larger.

### Fractional atomic coordinates and isotropic or equivalent isotropic displacement parameters (Å^2^)

**Table:**

	*x*	*y*	*z*	*U*_iso_*/*U*_eq_	
Cl1	0.78504 (18)	0.24214 (12)	0.34421 (3)	0.1169 (5)	
C3	0.7443 (5)	0.2768 (4)	0.24451 (11)	0.0695 (9)	
C4	0.7076 (5)	0.1802 (4)	0.20226 (12)	0.0744 (10)	
H4	0.5952	0.2084	0.1847	0.089*	
C5	0.8243 (6)	0.1400 (4)	0.12119 (10)	0.0775 (11)	
H5	0.7375	0.2071	0.1070	0.093*	
C1	0.7075 (6)	0.0576 (5)	0.27315 (13)	0.0840 (11)	
C2	0.7477 (5)	0.1979 (4)	0.28492 (11)	0.0773 (10)	
C6	1.0128 (6)	0.1530 (5)	0.09539 (12)	0.0901 (13)	
H6	1.0961	0.0856	0.1108	0.108*	
C10	0.7420 (7)	−0.0036 (4)	0.11707 (11)	0.1015 (13)	
H10A	0.6212	−0.0050	0.1327	0.122*	
H10B	0.8216	−0.0695	0.1338	0.122*	
C7	0.9896 (8)	0.1062 (5)	0.04242 (13)	0.1185 (18)	
H7A	0.9093	0.1714	0.0256	0.142*	
H7B	1.1099	0.1076	0.0265	0.142*	
C11	1.1050 (6)	0.2946 (6)	0.10072 (14)	0.1047 (15)	
H11	1.0932	0.3206	0.1350	0.126*	
C9	0.7204 (10)	−0.0480 (5)	0.06354 (14)	0.1288 (18)	
H9	0.6340	0.0161	0.0476	0.155*	
C8	0.9082 (10)	−0.0374 (7)	0.03860 (16)	0.140 (2)	
H8A	0.9932	−0.1035	0.0532	0.168*	
H8B	0.8942	−0.0616	0.0046	0.168*	
C14	1.0115 (8)	0.4096 (5)	0.07156 (16)	0.140 (2)	
H14A	1.0309	0.3936	0.0375	0.210*	
H14B	1.0641	0.4978	0.0804	0.210*	
H14C	0.8805	0.4100	0.0783	0.210*	
C13	1.3143 (7)	0.2870 (7)	0.08997 (15)	0.160 (2)	
H13A	1.3696	0.2151	0.1093	0.241*	
H13B	1.3712	0.3746	0.0977	0.241*	
H13C	1.3329	0.2667	0.0562	0.241*	
C12	0.6404 (13)	−0.1951 (7)	0.06041 (19)	0.217 (4)	
H12A	0.6598	−0.2313	0.0283	0.326*	
H12B	0.5096	−0.1925	0.0673	0.326*	
H12C	0.7017	−0.2537	0.0836	0.326*	
C18	0.7548 (7)	0.4780 (4)	0.18901 (13)	0.0971 (12)	
H18A	0.6422	0.4507	0.1721	0.117*	
H18B	0.8618	0.4566	0.1689	0.117*	
C15	0.7871 (6)	0.5142 (4)	0.27610 (13)	0.0967 (13)	
H15A	0.6884	0.5045	0.2999	0.116*	
H15B	0.9064	0.5059	0.2923	0.116*	
C17	0.7505 (7)	0.6280 (4)	0.20278 (19)	0.1103 (14)	
H17	0.7344	0.7001	0.1807	0.132*	
C16	0.7720 (7)	0.6461 (5)	0.2495 (2)	0.1092 (14)	
H16	0.7772	0.7329	0.2643	0.131*	
N1	0.7681 (5)	0.4111 (3)	0.23671 (9)	0.0791 (9)	
O3	0.8607 (3)	0.1789 (2)	0.17127 (7)	0.0767 (7)	
O1	0.6832 (4)	0.0450 (2)	0.22383 (8)	0.0845 (8)	
O2	0.6958 (5)	−0.0440 (3)	0.29863 (9)	0.1111 (10)	

### Atomic displacement parameters (Å^2^)

**Table:**

	*U*^11^	*U*^22^	*U*^33^	*U*^12^	*U*^13^	*U*^23^
Cl1	0.1428 (11)	0.1448 (9)	0.0630 (6)	0.0000 (9)	−0.0027 (6)	−0.0123 (6)
C3	0.057 (2)	0.086 (2)	0.065 (2)	0.002 (2)	0.0010 (19)	−0.009 (2)
C4	0.067 (3)	0.089 (3)	0.068 (2)	0.002 (2)	0.002 (2)	−0.0044 (19)
C5	0.084 (3)	0.096 (3)	0.053 (2)	0.012 (2)	−0.0074 (19)	−0.0018 (17)
C1	0.082 (3)	0.096 (3)	0.074 (3)	0.008 (3)	0.009 (2)	0.003 (2)
C2	0.072 (3)	0.100 (3)	0.061 (2)	0.005 (2)	0.001 (2)	−0.0071 (19)
C6	0.082 (3)	0.132 (4)	0.056 (2)	0.024 (3)	0.001 (2)	0.000 (2)
C10	0.131 (4)	0.107 (3)	0.066 (2)	−0.005 (3)	−0.007 (3)	−0.0180 (19)
C7	0.144 (5)	0.155 (5)	0.057 (2)	0.018 (4)	0.007 (3)	−0.011 (3)
C11	0.093 (4)	0.161 (5)	0.060 (2)	−0.010 (3)	0.009 (2)	−0.007 (3)
C9	0.182 (6)	0.129 (4)	0.075 (3)	−0.010 (4)	−0.010 (4)	−0.028 (3)
C8	0.193 (7)	0.155 (5)	0.071 (3)	0.011 (5)	0.012 (3)	−0.038 (3)
C14	0.165 (6)	0.146 (4)	0.110 (3)	−0.015 (4)	0.009 (4)	0.025 (3)
C13	0.098 (4)	0.294 (8)	0.088 (3)	−0.019 (5)	0.027 (3)	−0.011 (4)
C12	0.325 (10)	0.197 (6)	0.130 (4)	−0.106 (7)	0.031 (5)	−0.073 (4)
C18	0.097 (3)	0.098 (3)	0.097 (3)	0.007 (3)	0.010 (3)	0.010 (2)
C15	0.082 (3)	0.101 (3)	0.107 (3)	−0.010 (3)	−0.002 (3)	−0.022 (3)
C17	0.106 (4)	0.085 (3)	0.141 (4)	0.004 (3)	0.015 (4)	0.016 (3)
C16	0.088 (3)	0.088 (3)	0.152 (4)	−0.009 (3)	0.003 (3)	−0.021 (3)
N1	0.081 (3)	0.088 (2)	0.0686 (17)	0.0027 (19)	0.0051 (18)	−0.0100 (16)
O3	0.0764 (17)	0.1003 (17)	0.0532 (13)	0.0049 (14)	0.0025 (13)	−0.0071 (12)
O1	0.097 (2)	0.0833 (17)	0.0735 (15)	−0.0071 (15)	0.0035 (14)	−0.0038 (13)
O2	0.136 (3)	0.103 (2)	0.0939 (18)	0.016 (2)	0.0152 (19)	0.0119 (16)

### Geometric parameters (Å, °)

**Table:**

Cl1—C2	1.709 (3)	C9—C8	1.518 (7)
C3—N1	1.321 (4)	C9—C12	1.530 (7)
C3—C2	1.347 (4)	C9—H9	0.9800
C3—C4	1.512 (4)	C8—H8A	0.9700
C4—O3	1.393 (4)	C8—H8B	0.9700
C4—O1	1.441 (4)	C14—H14A	0.9600
C4—H4	0.9800	C14—H14B	0.9600
C5—O3	1.453 (3)	C14—H14C	0.9600
C5—C10	1.507 (5)	C13—H13A	0.9600
C5—C6	1.536 (5)	C13—H13B	0.9600
C5—H5	0.9800	C13—H13C	0.9600
C1—O2	1.206 (4)	C12—H12A	0.9600
C1—O1	1.375 (4)	C12—H12B	0.9600
C1—C2	1.418 (5)	C12—H12C	0.9600
C6—C11	1.522 (6)	C18—N1	1.466 (4)
C6—C7	1.535 (5)	C18—C17	1.492 (5)
C6—H6	0.9800	C18—H18A	0.9700
C10—C9	1.542 (5)	C18—H18B	0.9700
C10—H10A	0.9700	C15—C16	1.469 (6)
C10—H10B	0.9700	C15—N1	1.476 (4)
C7—C8	1.504 (6)	C15—H15A	0.9700
C7—H7A	0.9700	C15—H15B	0.9700
C7—H7B	0.9700	C17—C16	1.308 (5)
C11—C14	1.524 (6)	C17—H17	0.9300
C11—C13	1.535 (6)	C16—H16	0.9300
C11—H11	0.9800		
			
N1—C3—C2	133.1 (3)	C12—C9—H9	108.6
N1—C3—C4	119.9 (3)	C10—C9—H9	108.6
C2—C3—C4	107.0 (3)	C7—C8—C9	112.1 (5)
O3—C4—O1	109.9 (3)	C7—C8—H8A	109.2
O3—C4—C3	109.8 (3)	C9—C8—H8A	109.2
O1—C4—C3	105.0 (3)	C7—C8—H8B	109.2
O3—C4—H4	110.7	C9—C8—H8B	109.2
O1—C4—H4	110.7	H8A—C8—H8B	107.9
C3—C4—H4	110.7	C11—C14—H14A	109.5
O3—C5—C10	112.2 (3)	C11—C14—H14B	109.5
O3—C5—C6	105.0 (3)	H14A—C14—H14B	109.5
C10—C5—C6	112.8 (4)	C11—C14—H14C	109.5
O3—C5—H5	108.9	H14A—C14—H14C	109.5
C10—C5—H5	108.9	H14B—C14—H14C	109.5
C6—C5—H5	108.9	C11—C13—H13A	109.5
O2—C1—O1	119.6 (4)	C11—C13—H13B	109.5
O2—C1—C2	130.8 (3)	H13A—C13—H13B	109.5
O1—C1—C2	109.6 (3)	C11—C13—H13C	109.5
C3—C2—C1	110.1 (3)	H13A—C13—H13C	109.5
C3—C2—Cl1	130.7 (3)	H13B—C13—H13C	109.5
C1—C2—Cl1	119.2 (3)	C9—C12—H12A	109.5
C11—C6—C7	113.7 (4)	C9—C12—H12B	109.5
C11—C6—C5	114.4 (3)	H12A—C12—H12B	109.5
C7—C6—C5	108.6 (4)	C9—C12—H12C	109.5
C11—C6—H6	106.5	H12A—C12—H12C	109.5
C7—C6—H6	106.5	H12B—C12—H12C	109.5
C5—C6—H6	106.5	N1—C18—C17	101.4 (3)
C5—C10—C9	111.4 (3)	N1—C18—H18A	111.5
C5—C10—H10A	109.3	C17—C18—H18A	111.5
C9—C10—H10A	109.3	N1—C18—H18B	111.5
C5—C10—H10B	109.3	C17—C18—H18B	111.5
C9—C10—H10B	109.3	H18A—C18—H18B	109.3
H10A—C10—H10B	108.0	C16—C15—N1	102.0 (3)
C8—C7—C6	112.2 (4)	C16—C15—H15A	111.4
C8—C7—H7A	109.2	N1—C15—H15A	111.4
C6—C7—H7A	109.2	C16—C15—H15B	111.4
C8—C7—H7B	109.2	N1—C15—H15B	111.4
C6—C7—H7B	109.2	H15A—C15—H15B	109.2
H7A—C7—H7B	107.9	C16—C17—C18	112.1 (4)
C6—C11—C14	114.0 (4)	C16—C17—H17	123.9
C6—C11—C13	111.5 (5)	C18—C17—H17	123.9
C14—C11—C13	111.4 (5)	C17—C16—C15	112.5 (4)
C6—C11—H11	106.5	C17—C16—H16	123.7
C14—C11—H11	106.5	C15—C16—H16	123.7
C13—C11—H11	106.5	C3—N1—C18	124.5 (3)
C8—C9—C12	111.8 (5)	C3—N1—C15	123.3 (3)
C8—C9—C10	108.9 (4)	C18—N1—C15	111.7 (3)
C12—C9—C10	110.4 (4)	C4—O3—C5	116.2 (3)
C8—C9—H9	108.6	C1—O1—C4	108.2 (3)

### Hydrogen-bond geometry (Å, °)

**Table:**

*D*—H···*A*	*D*—H	H···*A*	*D*···*A*	*D*—H···*A*
C16—H16···O2^i^	0.93	2.42	3.320 (6)	164

Symmetry codes: (i) *x*, *y*+1, *z*.
